# Supplementary material for: The Geomagnetic Field (GMF) Is Required for Lima Bean Photosynthesis and Reactive Oxygen Species Production
Source: Int J Mol Sci. 2023 Feb 2;24(3):2896. doi: 10.3390/ijms24032896 (PMC9917513; doi:10.3390/ijms24032896)
Supplement: Supplementary file 1 [file ijms-24-02896-s001.zip › Supplementary Figure S1.pdf]

Supplementary Figure S1. OJIP Chlorophyll Fluorescence Induction Kinetics

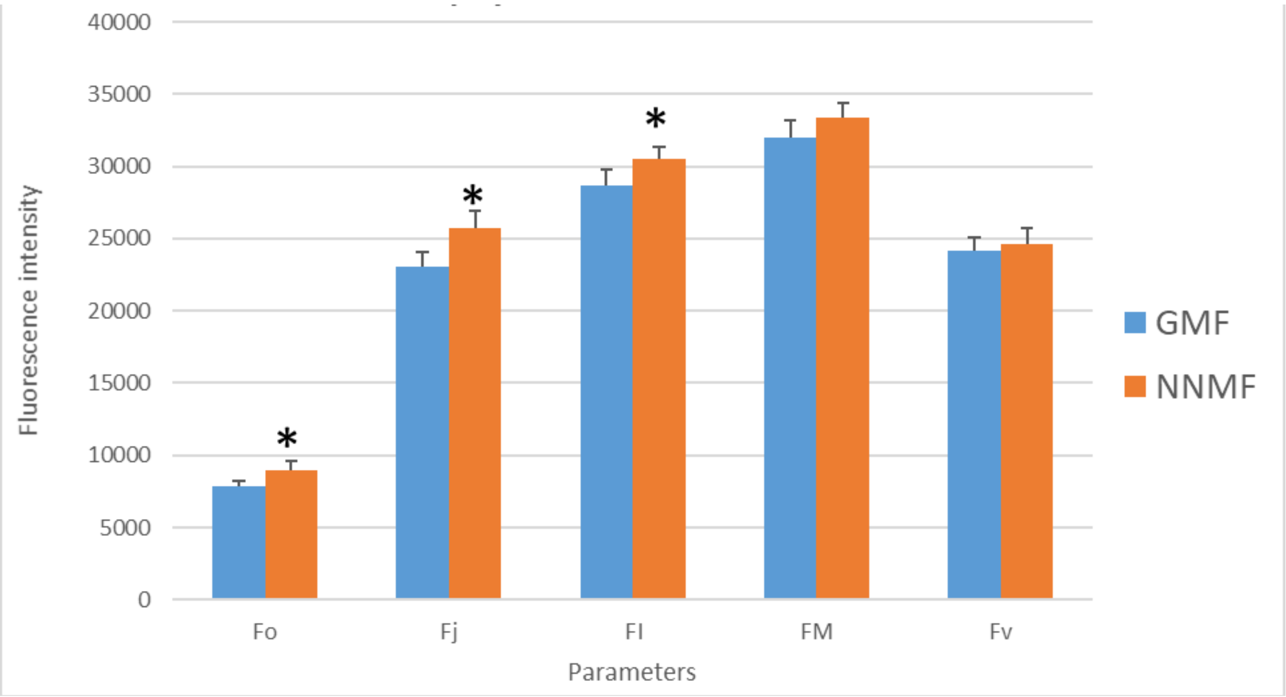

The initial fluorescence intensity (F_0), fluorescence intensity at about 2 ms (F_j) and at about 30 ms (F_I) of dark-adapted leaves are significantly higher in NNMF. Whereas maximal fluorescence level ($F_P = F_M$ under saturating excitation light) at 300 ms shows almost significant ($P = 0.065$) higher values in NNMF. The variable chlorophyll fluorescence ($F_M - F_o$) = Fv shows not significant differences between GMF and NNMF.
