# Supplementary material for: The Geomagnetic Field (GMF) Is Required for Lima Bean Photosynthesis and Reactive Oxygen Species Production
Source: Int J Mol Sci. 2023 Feb 2;24(3):2896. doi: 10.3390/ijms24032896 (PMC9917513; doi:10.3390/ijms24032896)
Supplement: Supplementary file 1 [file ijms-24-02896-s001.zip › Supplementary Figure S2.pdf]

Supplementary Figure S2. Non photochemical quenching.

Dark adapted leaves were exposed to actinic light as reported in the figure (yellow bar) followed by dark recovery (gray bar). Asterisks indicate significant differences between GMF and NNMF values.

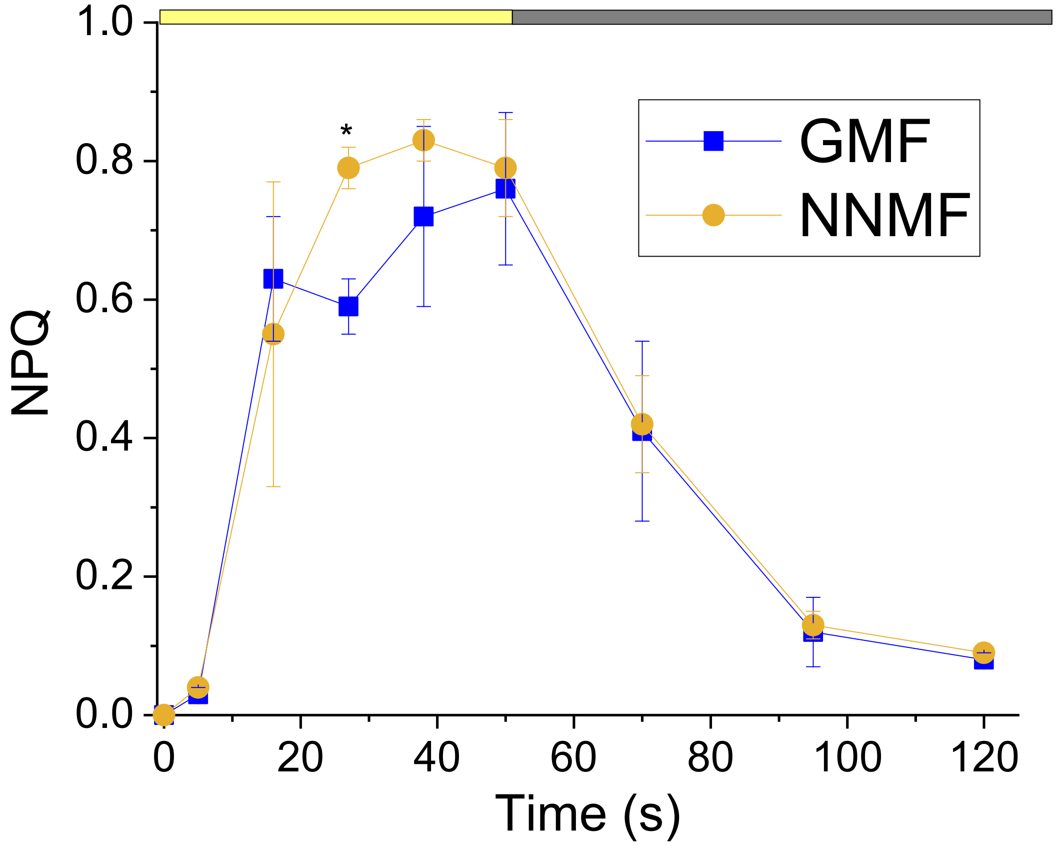

Phase	Duration	# of pulses	1st pulse	Pulse interval
Light	60 s	5	7 s	12 s
Dark recovery	88 s	3	11 s	26 s

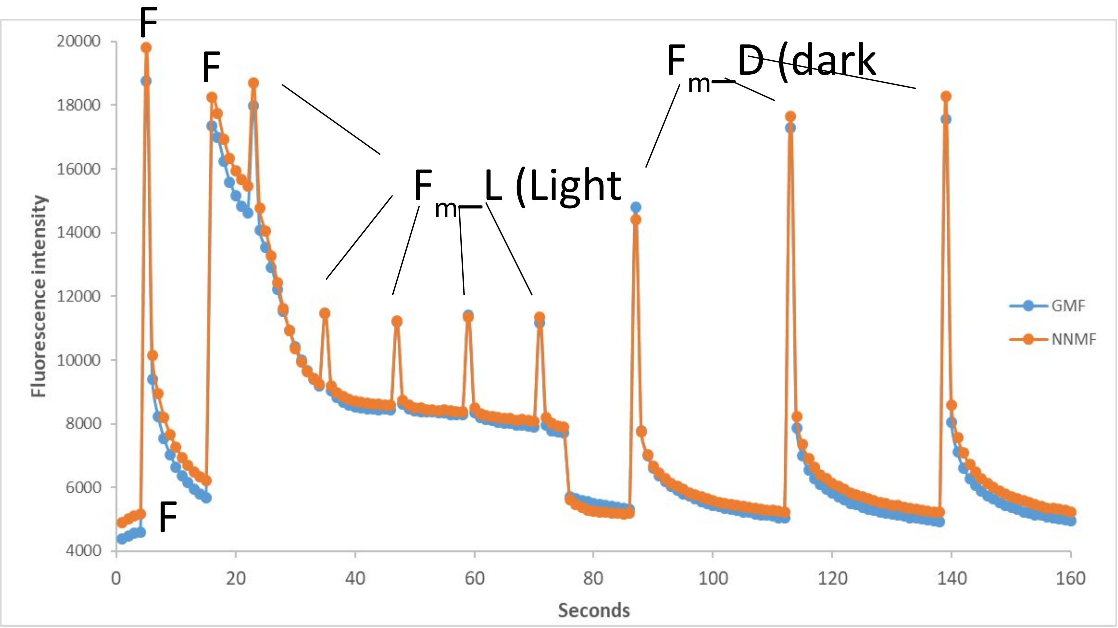

	GMF		NNMF		ANOVA	
	Mean	SD	Mean	SD	F-ratio	P value
**Fp**	17534.17	333.91	**18267.33**	**529.38**	**8.986**	**0.010**
**Fm_L1**	18335.80	199.06	**18981.29**	**419.47**	**10.008**	**0.010**
Fm_L2	12849.50	2300.97	12592.00	1760.34	0.041	0.845
Fm_L3	11911.80	521.57	11931.20	995.95	0.010	0.970
Fm_L4	11339.60	683.63	11053.40	378.99	0.670	0.437
Fm_Lss	10929.80	627.51	10897.40	237.14	0.012	0.917
NPQ_L1	0.03	0.01	0.04	0.01	3.173	0.095
NPQ_L2	0.63	0.09	0.55	0.22	0.515	0.489
**NPQ_L3**	0.59	**0.04**	**0.79**	**0.03**	**70.620**	**0.000**
NPQ_L4	0.72	0.13	0.83	0.03	1.934	0.214
NPQ_Lss	0.76	0.11	0.79	0.07	0.394	0.548
Qp_L1	0.19	0.06	0.21	0.03	0.679	0.431
Qp_L2	0.26	0.06	0.25	0.06	0.112	0.743
Qp_L3	0.34	0.07	0.34	0.06	0.015	0.905
Qp_L4	0.39	0.06	0.38	0.05	0.081	0.782
Qp_Lss	0.53	0.09	0.53	0.06	0.394	0.548
**Rfd**	1.16	**0.10**	**1.30**	**0.06**	**6.787**	**0.031**
Fm_D1	13901.60	827.95	13957.67	664.11	0.016	0.903
**Fm_D2**	17067.60	**383.30**	**17458.13**	**294.02**	**4.327**	**0.062**
**Fm_D3**	17575.20	**170.02**	**18186.20**	**153.29**	**35.618**	**0.000**
NPQ_D1	0.41	0.13	0.42	0.07	0.065	0.805
NPQ_D2	0.12	0.05	0.13	0.02	0.438	0.521
NPQ_D3	0.08	0.01	0.09	0.01	1.697	0.217
Qp_D1	0.85	0.04	0.87	0.05	0.587	0.461
Qp_D2	0.93	0.02	0.93	0.03	0.205	0.659
**Qp_D3**	0.94	**0.02**	**0.97**	**0.01**	**11.391**	**0.006**
QY_max	0.78	0.01	0.76	0.02	2.623	0.140
QY_L1	0.18	0.04	0.15	0.02	1.642	0.232
QY_L2	0.17	0.03	0.18	0.02	0.365	0.561
QY_L3	0.22	0.05	0.21	0.04	0.322	0.583
QY_L4	0.25	0.04	0.24	0.03	0.118	0.739
QY_Lss	0.26	0.05	0.26	0.04	0.010	0.924
QY_D1	0.62	0.03	0.62	0.02	0.141	0.716
QY_D2	0.70	0.01	0.70	0.01	0.882	0.362
QY_D3	0.72	0.00	0.72	0.00	0.021	0.887
