# Supplementary material for: The Geomagnetic Field (GMF) Is Required for Lima Bean Photosynthesis and Reactive Oxygen Species Production
Source: Int J Mol Sci. 2023 Feb 2;24(3):2896. doi: 10.3390/ijms24032896 (PMC9917513; doi:10.3390/ijms24032896)

Supplementary Figure S3.

Pmf measurements on chlorophylls basis in Lima bean leaves grown in GMF and NNMF conditions. Measurements were conducted upon exposure to different light intensities. Data are means of five biological replicates with standard deviation shown and have been normalized to GMF sample exposed to 1200 μmol $m^{-2}s^{-1}$ of light.

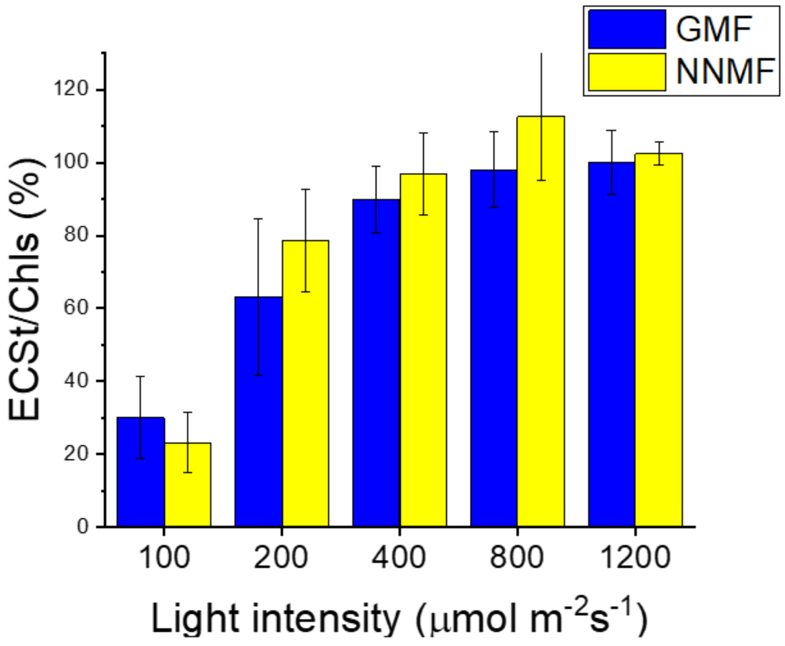

Supplement: Supplementary file 1 [file ijms-24-02896-s001.zip › Supplementary Figure S3.pdf]
