# Supplementary material for: The Geomagnetic Field (GMF) Is Required for Lima Bean Photosynthesis and Reactive Oxygen Species Production
Source: Int J Mol Sci. 2023 Feb 2;24(3):2896. doi: 10.3390/ijms24032896 (PMC9917513; doi:10.3390/ijms24032896)
Supplement: Supplementary file 1 [file ijms-24-02896-s001.zip › Supplementary Figure S4.pdf]

Supplementary Figure S4

Time-course variations of the magnetic field intensity B expressed as nT inside the triaxial Helmholtz coils system with current (i.e., NNMF) and without current (i.e., GMF) in the coils. Data are representative of 24 h. As it can be seen, no other sources of MF were present but the GMF.

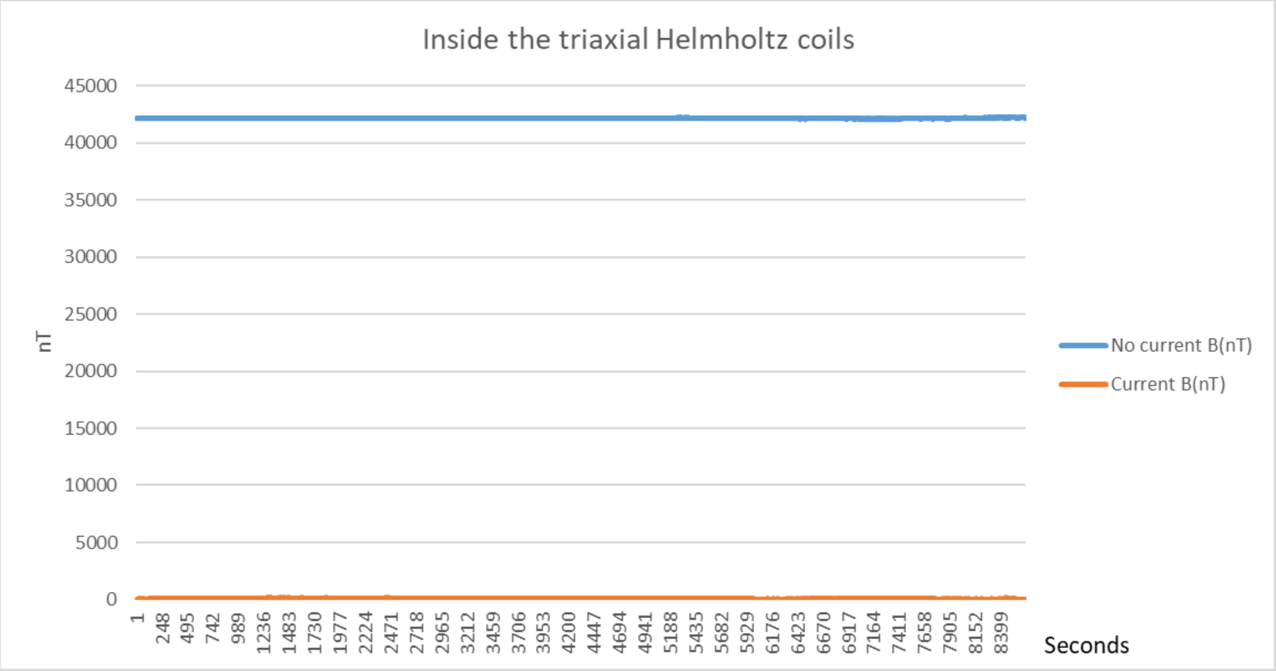

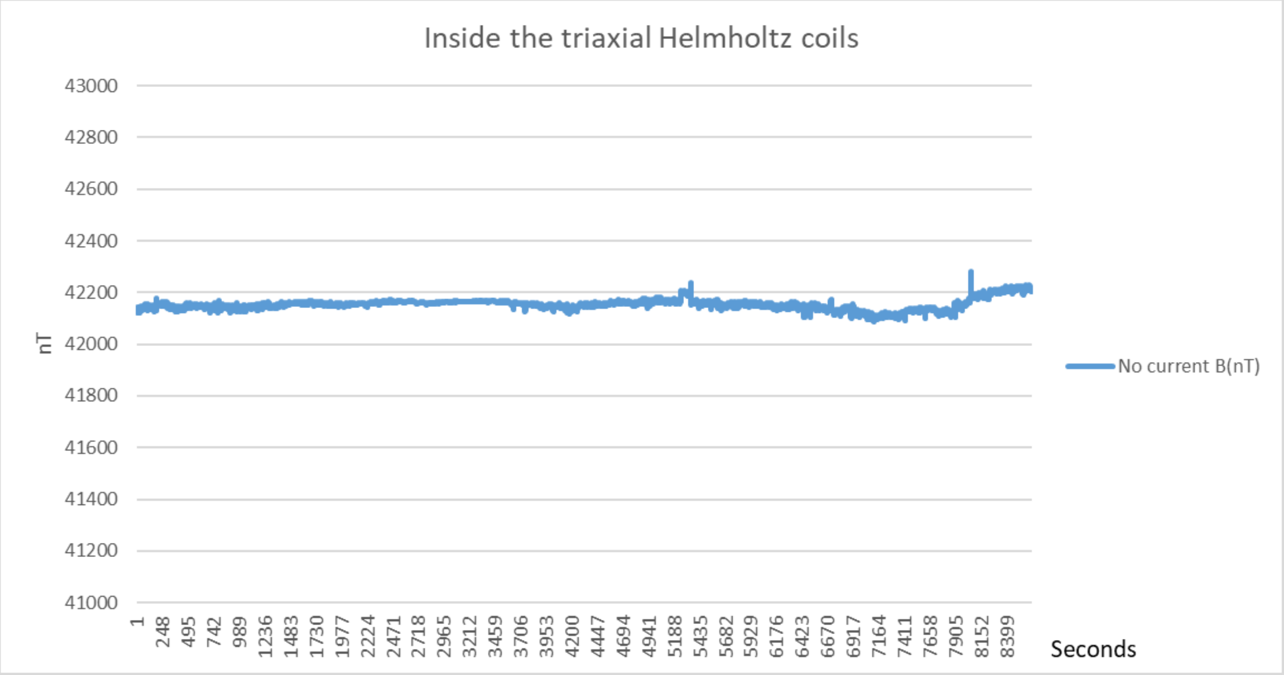

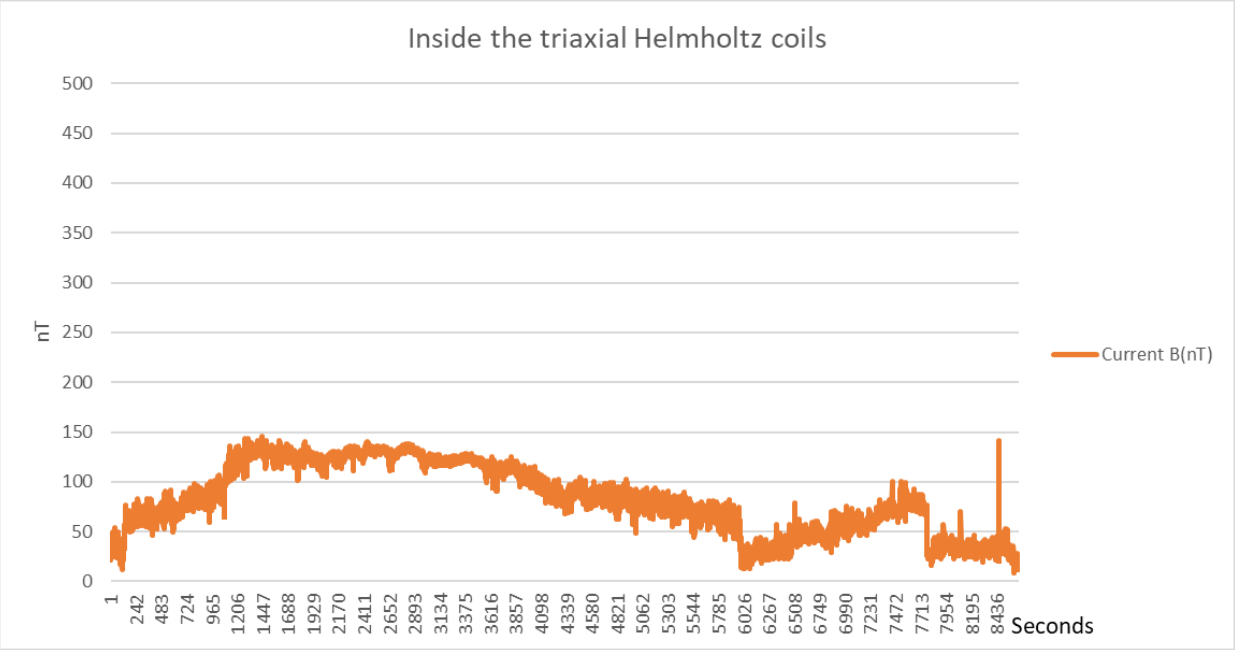
Inside the triaxial Helmholtz coils
nT
500
450
400
350
300
250
200
150
100
50
0
1
242
483
724
965
1206
1447
1688
1929
2170
2411
2652
2893
3134
3375
3616
3857
4098
4339
4580
4821
5062
5303
5544
5785
6026
6267
6508
6749
6990
7231
7472
7713
7954
8195
8436
Seconds
Current B(nT)
