# Supplementary material for: The Geomagnetic Field (GMF) Is Required for Lima Bean Photosynthesis and Reactive Oxygen Species Production
Source: Int J Mol Sci. 2023 Feb 2;24(3):2896. doi: 10.3390/ijms24032896 (PMC9917513; doi:10.3390/ijms24032896)
Supplement: Supplementary file 1 [file ijms-24-02896-s001.zip › Supplementary Table S1.pdf]

Supplementary Table S1. Primers used in this study

Gene	Locus	Sequence 5′ → 3′	Sense
PlIScA1	Pl01G0000340900.1	AGTTGGTTGAGGATAAGGGTGT	Forward
PlIScA1	Pl01G0000340900.1	CACCACAACCACACTGACCTT	Reverse
PlIScA2	Pl03G0000156300.1	TCACGATTCCGACACAACCA	Forward
PlIScA2	Pl03G0000156300.1	TTTTCGAACGCAGTTGTGGG	Reverse
PlcpIScA	Pl02G0000398800.1	ACGGATAACGCACTGAAGCA	Forward
PlcpIScA	Pl02G0000398800.1	GACATACCAGAGCACCCACC	Reverse
PlUBP6	Pl10G0000334000.1	TGGCTGGGTCAAGCAAGAAA	Forward
PlUBP6	Pl10G0000334000.1	ACGCCATATGCCAATCACCT	Reverse
SOD	DQ159910	AAGCACCACAGGGCTTATGT	Forward
SOD	DQ159910	ATGGTTTTCCACCTCCACCT	Reverse
CAT	DQ004737	GCCGCATGGTCTTGAATAAG	Forward
CAT	DQ004737	ATTTGGGTGCATTAGCAGGA	Reverse
APX	DQ004738	AGGAGCGTTCTGGATTTGAG	Forward
APX	DQ004738	AATCAGCGAAGAACGCATCT	Reverse
PRX	DQ159909	GACCCTGTCATGGACCAAAC	Forward
PRX	DQ159909	CAATGCCCTTGGTCCTCTTA	Reverse
GR	DQ159906	GCCCCTTCATTCCTGATATTC	Forward
GR	DQ159906	TCGTCAAATCCCCTCAGAAC	Reverse
GPX	DQ004739	AACAGGAAACTCAGCCTTGAA	Forward
GPX	DQ004739	ATTGTCAACGTTGCCTCACA	Reverse
PlActin1	DQ159907	AGGCTCCTCTTAACCCCAAG	Forward
PlActin1	DQ159907	GTGGGAGAGCATAACCCTCA	Reverse
