# Supplementary material for: The Geomagnetic Field (GMF) Is Required for Lima Bean Photosynthesis and Reactive Oxygen Species Production
Source: Int J Mol Sci. 2023 Feb 2;24(3):2896. doi: 10.3390/ijms24032896 (PMC9917513; doi:10.3390/ijms24032896)
Supplement: Supplementary file 1 [file ijms-24-02896-s001.zip › Supplementary Table S2.pdf]

Supplementary Table S2. Chromatographic gradient used for the separation of Chlorophyll, chlorophyll degradation products, and carotenoids.

Time (min)	Solvent A (%)	Solvent B (%)
0	95	5
5	60	40
10	50	50
12	48	52
14	45	55
16	40	60
18	40	60
20	20	80
22	20	80
24	5	95
27	5	95
27.01	95	5
30	95	5
0	95	5
